# Emergency Department Recruitment and Screening Strategies from Public and Private Hospitals in Brazil

**DOI:** 10.1002/alz70858_100875

**Published:** 2025-12-25

**Authors:** Thiago J Avelino‐Silva

**Affiliations:** ^1^ University of Sao Paulo Medical School, Sao Paulo, Sao Paulo, Brazil; University of California, San Francisco, San Francisco, CA, USA

## Abstract

Older adults presenting to emergency departments (EDs) routinely undergo delirium assessments, yet many also live with pre‐existing cognitive impairment that standard approaches may miss. Recognizing and examining these impairments in the ED is important, as this care setting often represents a key point where timely detection of unrecognized deficits can influence clinical decisions, hospital outcomes, and transitions of care. Drawing on our experience at both a large public hospital (Hospital das Clínicas, University of São Paulo) and a leading private hospital (Hospital Sírio‐Libanês) in Brazil, we will discuss practical strategies that supported participant recruitment across diverse socioeconomic backgrounds—ranging from adapted consent procedures to specialized training for research staff—to accommodate the fast‐paced nature of emergency care. While the public institution navigated high patient volume, limited resources, and complex patient flow, the private setting offered a more resource‐intensive environment with different logistical considerations. Comparing our experiences illustrates how variations in patient demographics, staffing structures, and institutional culture can shape both the feasibility and consistency of ED‐based studies. We will also address ethical and practical considerations around enrolling individuals with cognitive impairment, including capacity to consent, surrogate involvement, and minimizing patient burden. We then will describe how we employed the brief Confusion Assessment Method (bCAM) and the 10‐Point Cognitive Screener (10‐CS) to distinguish delirium from underlying impairment, identifying many cases of previously unrecognized cognitive challenges. Integrating these tools within our study protocols frequently revealed deficits that might have remained undetected using routine assessments, reflecting the hidden prevalence of such conditions in the ED. Our experiences demonstrate the need to identify and include older adults with dementia or undiagnosed cognitive impairment in ED research, offering suggestions on how enrollment and assessment strategies can be refined to encourage early recognition, guide risk stratification, and improve outcomes for vulnerable populations. Ultimately, our work shows how systematically combining inclusive recruitment with brief, efficient screening can expand study representativeness and inform targeted interventions for older adults who may be at higher risk.

